# Rescue of Dystrophic Skeletal Muscle by PGC-1α Involves a Fast to Slow Fiber Type Shift in the mdx Mouse

**DOI:** 10.1371/journal.pone.0030063

**Published:** 2012-01-11

**Authors:** Joshua T. Selsby, Kevin J. Morine, Klara Pendrak, Elisabeth R. Barton, H. Lee Sweeney

**Affiliations:** 1 Department of Physiology, School of Medicine, University of Pennsylvania, Philadelphia, Pennsylvania, United States of America; 2 Department of Animal Science, College of Agriculture and Life Sciences, Iowa State University, Ames, Iowa, United States of America; 3 Department of Anatomy and Cell Biology, School of Dental Medicine, University of Pennsylvania, Philadelphia, Pennsylvania, United States of America; Universidade Federal do Rio de Janeiro, Brazil

## Abstract

Increased utrophin expression is known to reduce pathology in dystrophin-deficient skeletal muscles. Transgenic over-expression of PGC-1α has been shown to increase levels of utrophin mRNA and improve the histology of mdx muscles. Other reports have shown that PGC-1α signaling can lead to increased oxidative capacity and a fast to slow fiber type shift. Given that it has been shown that slow fibers produce and maintain more utrophin than fast skeletal muscle fibers, we hypothesized that over-expression of PGC-1α in post-natal mdx mice would increase utrophin levels via a fiber type shift, resulting in more slow, oxidative fibers that are also more resistant to contraction-induced damage. To test this hypothesis, neonatal mdx mice were injected with recombinant adeno-associated virus (AAV) driving expression of PGC-1α. PGC-1α over-expression resulted in increased utrophin and type I myosin heavy chain expression as well as elevated mitochondrial protein expression. Muscles were shown to be more resistant to contraction-induced damage and more fatigue resistant. Sirt-1 was increased while p38 activation and NRF-1 were reduced in PGC-1α over-expressing muscle when compared to control. We also evaluated if the use a pharmacological PGC-1α pathway activator, resveratrol, could drive the same physiological changes. Resveratrol administration (100 mg/kg/day) resulted in improved fatigue resistance, but did not achieve significant increases in utrophin expression. These data suggest that the PGC-1α pathway is a potential target for therapeutic intervention in dystrophic skeletal muscle.

## Introduction

Dystrophin is a structural protein linking cytoskeletal actin to the sarcolemma through the dystrophin-glycoprotein complex. Duchenne muscular dystrophy (DMD) is an X-linked, progressive muscle wasting disease caused by a non-functional dystrophin protein [1]. Clinically, this disease is normally diagnosed in the pre-school years as early developmental milestones are missed. It then progresses rapidly to wheelchair confinement by the early teen years followed by death due to respiratory or cardiac failure by the third decade. Whole muscles become progressively more fibrotic as greater numbers of fibers are lost to necrosis, impairing muscle function.

The primary functional defect resulting from dystrophin deficiency appears to be an increased susceptibility to contraction-induced rupture of the sarcolemma [2]. These sarcolemmal lesions, and possibly leaky Ca^2+^ channels [3], [4], increase Ca^2+^ influx into dystrophic fibers leading to protease activation [5], [6], [7] and free radical formation via cytosolic [8], [9], [10] and mitochondrial sources [11], [12], [13]. Excess Ca^2+^ is sequestered first in the sarcoplasmic reticulum (SR) followed by the mitochondria, potentially contributing to pathologies in both organelles. Indeed, increased Ca^2+^ content in the SR and mitochondria has been detected in dystrophic skeletal muscle [14], [15]. Moreover, impaired ATP production and metabolic abnormalities have also been reported [16], [17].

Upregulation of the dystrophin-related protein, utrophin, is among the most promising of potential strategies for the treatment of DMD [18]. Utrophin is a structural protein that shares many similarities with dystrophin including actin and glycoprotein binding domains as well as hinge regions and spectrin-like repeats. Utrophin over-expression has been shown to rescue dystrophic skeletal muscles from dystrophin-deficient mouse [19], [20], [21] and dog models [22]. In mature skeletal muscle fibers utrophin expression is primarily limited to the nuclei at neuromuscular junctions, however, it is more widely expressed during regeneration [23]. Utrophin expression is under transcriptional [24], as well as post-transcriptional control [25], and levels of utrophin A gene expression are 3–4 fold higher in slow fiber types than in fast fibers due to both transcriptional and post-transcriptional modulation [26].

Given the apparent importance of utrophin upregulation as a therapy for DMD it is critical to establish a strategy leading to increased utrophin expression. Utrophin gene expression may be induced by a complex that associates peroxisome proliferator-activated receptor gamma coactivator 1-alpha (PGC-1α) and GABP via Host Cell Factor (HCF) or neuregulin, acting on the N-box domain of the utrophin gene [27], [28], [29].

Development of therapeutic strategies designed to offset mitochondrial dysfunction experienced in dystrophic skeletal muscle is also a high priority. Through a PGC-1α/ERRα/NRF-1/MTFa pathway, PGC-1α can also drive oxidative gene expression, potentially aiding damaged mitochondria and supporting ATP production [28], [30], [31], [32], [33]. Regulation of these divergent PGC-1α pathways appears to be phosphorylation dependent where unphosphorylated PGC-1α may favor induction of oxidative genes and MAPK p38 phosphorylation of PGC-1α and/or GABP drives fate toward slow gene expression [27]. It was previously shown that dystrophin-deficient mice with transgenic PGC-1α over-expression had reduced muscle injury in both sedentary and exercised conditions [27]. Many of these effects can be attributed to increased utrophin expression and increased oxidative capacity [27]. Despite this early success, it must be recognized that there may be significant therapeutic benefits during pre-natal development that would not be translatable to human populations [34], [35]. Consequently, there is a need to determine the extent to which PGC-1α over-expression following birth will attenuate the dystrophic phenotype.

Further, PGC-1α is activated by a number of upstream pathways including activation of Sirt-1 and AMPK. Sirt-1 activation increases PGC-1α activity through deacetylation and may be activated through resveratrol exposure [36], [37], [38]. Therefore resveratrol, or more potent Sirt-1 activators [39], may provide a pharmacological means to increase PGC-1α signaling in both mdx mice and eventually in DMD patients. Accordingly, we also examined the potential of resveratrol to mimic the effects of PGC-1α over-expression.

## Results

**Figure 1:**
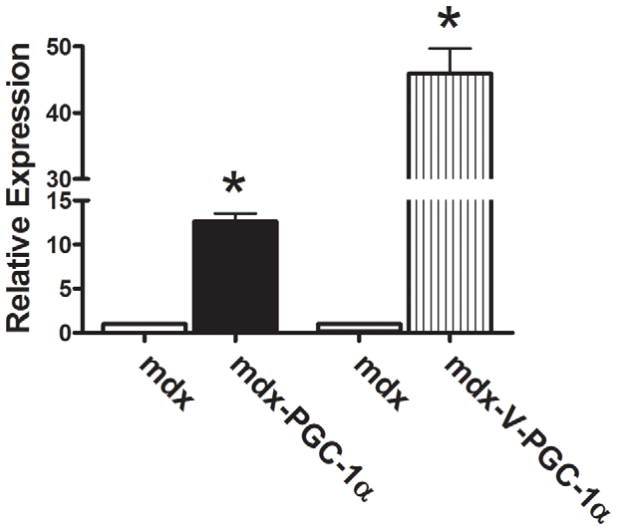
Virally-mediated gene transfer. Six weeks following PGC-1α gene delivery total (PGC-1α) and viral (V-PGC-1α) expression was increased in treated limbs compared to untreated limbs (n = 6/group). * indicates p<0.05.

PGC-1α expression was found to be on average 12-fold higher in treated limbs when compared to control, with a range of 9.6–14.5 fold increase (Figure 1). Over-expression was due to exogenous PGC-1α produced by our vector, as confirmed using PCR primers against the 3′ UTR from our viral construct.

### Muscle function (4 and 6 week old animals)

On the whole, muscle masses were reduced in the limbs over-expressing PGC-1α compared to control injected limbs at both four and six weeks (Table 1). At four weeks of age the TA, EDL, and gastrocnemius were reduced approximately 10%, 10%, 26%, respectively (p<0.05), and the soleus was similar between groups. At six weeks the masses of the TA, EDL, gastrocnemius, and soleus were reduced by approximately 20%, 15%, 27%, and 12%, respectively (p<0.05).

**Table 1:** PGC-1α induced changes in muscle mass.

	*EDL (mg)*	*Soleus (mg)*	*Gastroc (mg)*	*TA (mg)*
4 Week mdx	5.3±0.3	3.7±0.1	62.0±3	22.4±2
4 Week mdx-PGC-1α	4.8±0.5*	3.6±0.2	45.8±4*	20.0±2*
6 Week mdx	9.9±0.5	8.8±0.6	131.9±6	51.3±2
6 Week mdx-PGC-1α	8.4±0.5*	7.8±0.6*	96.4±6*	41.6±2*

Following four (n = 7) or six (n = 13) weeks of PGC-1α over-expression in mdx mice muscle mass was generally reduced.

*indicates significantly different from corresponding control. EDL – extensor digitorum longus, Gastroc – gastrocnemius, TA – tibialis anterior.

The EDL and soleus were further examined for muscle function. Due to the reduction in muscle mass, there was a corresponding reduction in muscle CSA in both the soleus and EDL at four and six weeks (Table 2). Despite the reductions in muscle mass and CSA, tetanic force was similar for both muscles at four weeks and at six weeks. Thus, despite their smaller size, muscles over-expressing PGC-1α were able to maintain force comparable, if not greater than control muscles. Finally, PGC-1α over-expression caused a 12% increase in specific tension in the six week EDL.

**Table 2:** PGC-1α induced changes in muscle function.

	*CSA (mm^2^)*	*Tetanic Force (mN)*	*Specific Tension (N/cm^2^)*
4 wk mdx sol	0.62±0.03	55.4±5	9.1±0.8
4 wk mdx-PGC-1α sol	0.57±0.04*	61.7±6	10.7±1.2
4 wk mdx EDL	1.1±0.05	156±9	14±0.6
4 wk mdx-PGC-1α EDL	1.0±0.08*	155±13	15.5±0.8
6 wk mdx sol	0.97±0.05	142±6	14.6±0.5
6 wk mdx-PGC-1α sol	0.87±0.05*	119±11	13.7±1.0
6 wk mdx EDL	1.7±0.06	292±23	16.8±1.0
6 wk mdx-PGC-1α EDL	1.5±0.06*	289±15	19.1±1.0*

Following four (n = 7) or six (sol n = 6; EDL n = 13) weeks of PGC-1α over-expression muscle function in the soleus and EDL was altered.

*indicates significantly different from corresponding control. Wk – week, sol – soleus, EDL – extensor digitorum longus, CSA – cross sectional area.

**Figure 2:**
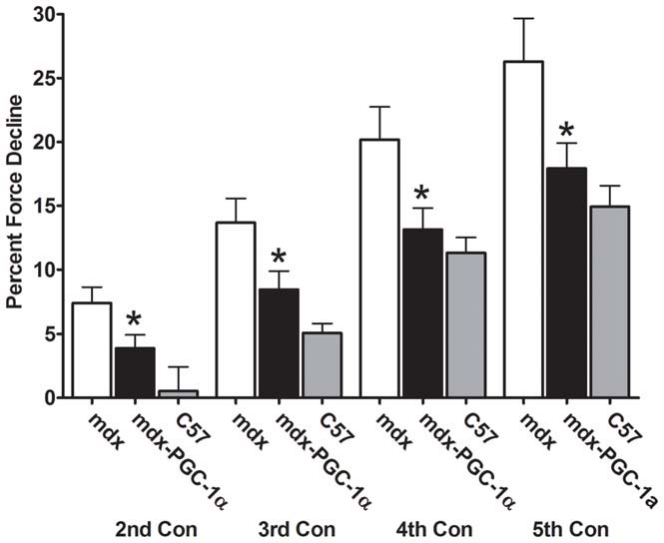
Relative force reduction in 6 wk old EDL's following lengthening contractions. PGC-1α over-expressing limbs were better able to maintain force during lengthening contractions when compared to control limbs (n = 13/group). Data from age matched C57 animals is included for reference purposes. * indicates p<0.05. Con – contraction.

**Figure 3:**
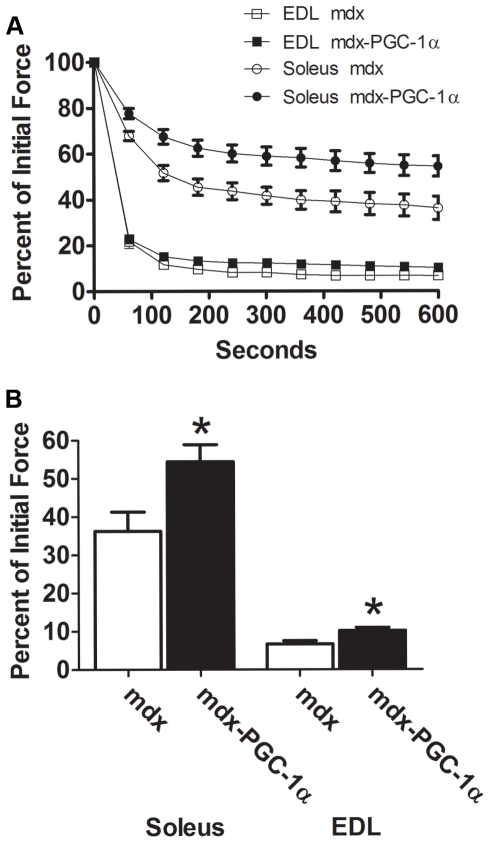
PGC-1α protects against muscle fatigue. Muscle fatigue curves in the soleus (n = 6/group) and EDL (n = 7/group) (A) during 10 minutes of a fatigue protocol where muscles are contracted every second for 10 minutes. Force of the final contraction (B) was significantly higher in the soleus and EDL muscles over-expressing PGC-1α when compared to control muscle. * indicates p<0.05.

**Figure 4:**
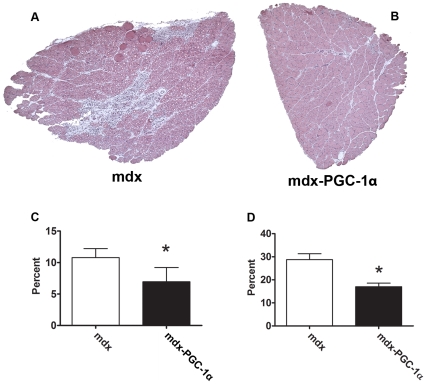
PGC-1α over-expression reduces disease-related muscle injury. 10× micrographs of six week old soleus muscles following H and E staining (A and B). (C) The total areas of necrotic, H&E negative, or regenerating cells were quantified and is expressed as a percent of the total soleus area. In addition, laminin was detected with a fluorescently labeled antibody (not shown) in order to determine central nucleation. (D) PGC-1α caused a reduction in central nucleation. N = 5/group; * indicates p<0.05.

To determine the extent to which PGC-1α improved resistance to contraction induced injury, muscles underwent a series of five lengthening contractions. Forces were scaled to the maximal force generated during the first contraction. PGC-1α over-expression allowed a maintenance of 30–40% (p<0.05) greater force output during each contraction in the EDL (Figure 2). PGC-1α over-expression failed to improve resistance to contraction induced injury in the soleus (Figure S1). In order to determine the extent to which increasing PGC-1α would improve fatigue resistance, an endurance challenge was given to the muscles. Muscles were sub-maximally stimulated every second for ten minutes. The relative force generated during the final contraction was approximately 65% greater in the soleus (p<0.05) and 34% greater in the EDL (p<0.05) from six week old muscles over-expressing PGC-1α compared to control limbs (Figure 3). This pattern was also observed in the soleus taken from four week old animals (Figure S2). Importantly, muscle mass was not well correlated with fatigue resistance indicating changes observed are the result of improved physiology, not better oxygen diffusion in smaller muscles (Figure S3). Finally, PGC-1α over-expression reduced damage and centralized nucleation (indicator of ongoing degeneration/regeneration) in soleus muscles (Figure 4). Fiber area distribution was similar between groups (Figure S4).

### Protein expression (6 week old animals)

**Figure 5:**
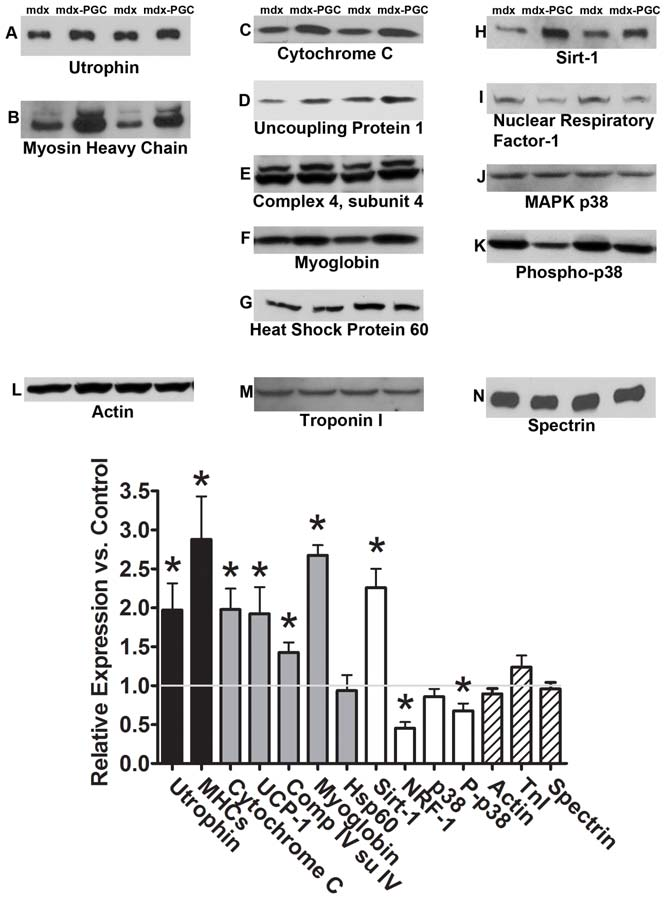
Protein expression following 6 weeks of PGC-1α over-expression. Slow genes are shown in black (utrophin – A, slow myosin heavy chain – B) oxidative genes are shown in gray (cytochrome C – C, uncoupling protein-1 – D, complex IV subunit IV – E, myoglobin – F, Hsp 60 – G), pathway genes are shown in white (Sirt-1 – H, nuclear respiratory factor -1 – I, p38 – J, phospho-p38 – K), and controls have diagonal lines (Actin – L, Troponin – M, Spectrin – N). Relative change compared to control limbs (n = 6/group) (O). * indicates p<0.05 compared to control limbs.

As the overall condition and function of muscles over-expressing PGC-1α was improved, we sought to determine the extent to which PGC-1α over-expression induced expression of utrophin and other proteins related to slow muscle. In order to maximize data produced from these animals, Western blots were performed in gastrocnemius with limited confirmation in the soleus. Western blot analysis revealed that utrophin expression was increased two fold in the gastrocnemius (p<0.05; Figure 5) and approximately 17% in the soleus (Figure S5; p<0.05) in response to PGC-1α over-expression. To confirm that the increase in utrophin was due to an absolute increase in protein, the membrane protein spectrin and the regulatory protein troponin were evaluated. In both cases, they were similar between groups indicating that the increase in utrophin is due to protein accumulation rather than merely a reflection of smaller fibers with increased surface area to volume ratios.

**Figure 6:**
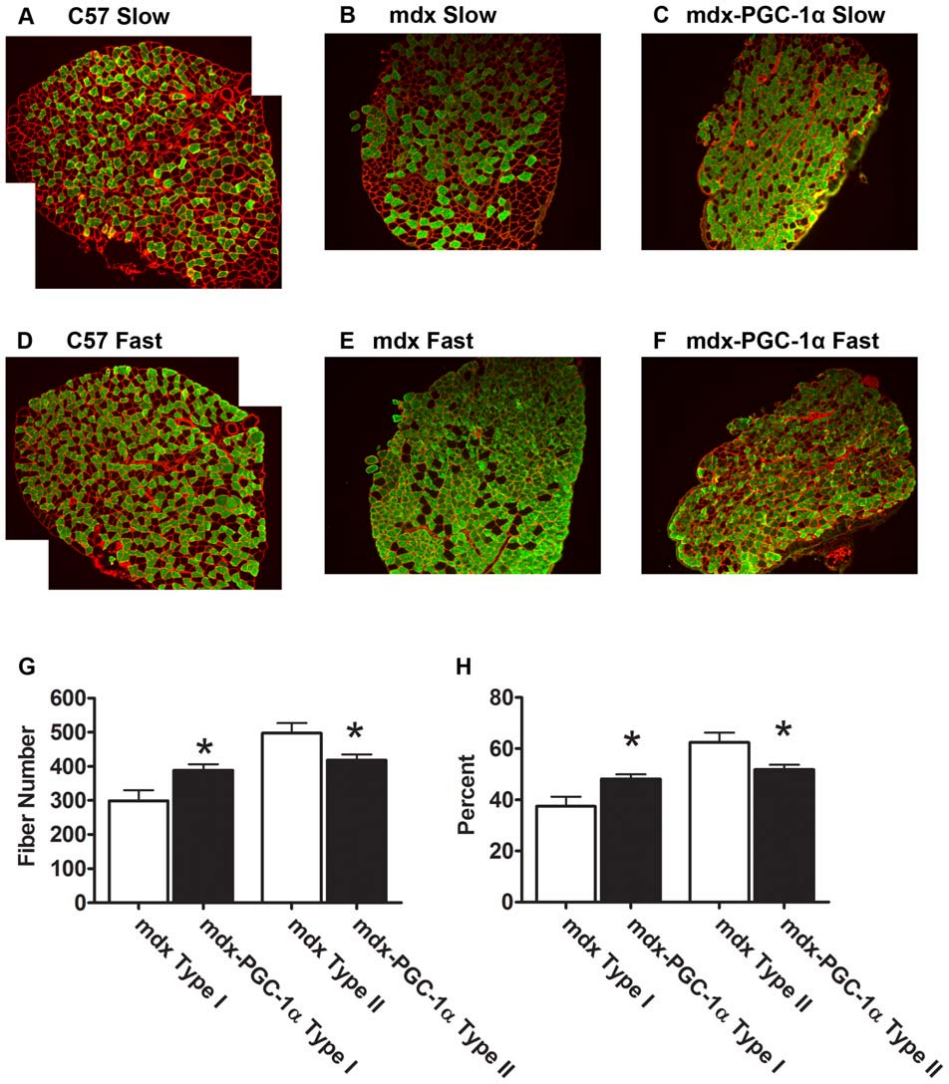
Myosin fiber type distribution in treated and control soleus muscles. Histological sections of soleus (10×) were exposed to an antibody against the slow isoform of myosin heavy chain (A). A C57 (healthy) section is included as reference (Left). The control limb (middle) shows moderate type I content. The corresponding injected limb (Right) clearly has an elevation in type I content. In serial sections, we also evaluated type II myosin heavy chain content (B). A C57 (healthy) section is included for reference (Left). The control limb (middle) shows high levels of type II expression. PGC-1α caused a reduced expression of type II fibers (Right). The absolute fiber numbers from each section expressing type I and II fibers were counted and recorded (C). PGC-1α caused a shift toward type I fibers and away from type II fibers. These were then made relative to total fiber number (D). Once again, a type I shift is observed. N = 9/group; * indicates p<0.05.

Slow myosin heavy chain was increased by three fold in PGC-1α over-expressing muscle compared to control (Figure 5). We confirmed these data by quantifying the fiber type switch occurring in the soleus through histological determination of type I and type II fibers (Figure 6). Consistent with the Western blot analysis, an increase in the total number and relative number of type I staining fibers was detected as well as a corresponding decrease in type II fibers.

PGC-1α has also been implicated in mitochondrial biogenesis. As it was apparent that muscles were more fatigue resistant we assessed several oxidative and mitochondrial markers. Cytochrome C, complex IV subunit IV, myoglobin, and UCP-1 were increased between 40% and 2.5 fold (p<0.05) in PGC-1α over-expressing muscles while Hsp 60 remained unchanged when compared to control limbs (Figure 5).

Finally, we made initial determinations of several PGC-1α pathway activity regulators. Upstream activator Sirt-1 was increased 2.25 fold, however, an alternative activator, phospho-p38, was reduced by 33% in treated limbs when compared to control limbs with p38 similar between groups (Figure 5). Finally, NRF-1 was decreased by 50% in PGC-1α over-expressing muscle when compared to control muscle.

### 6 mo animals

**Figure 7:**
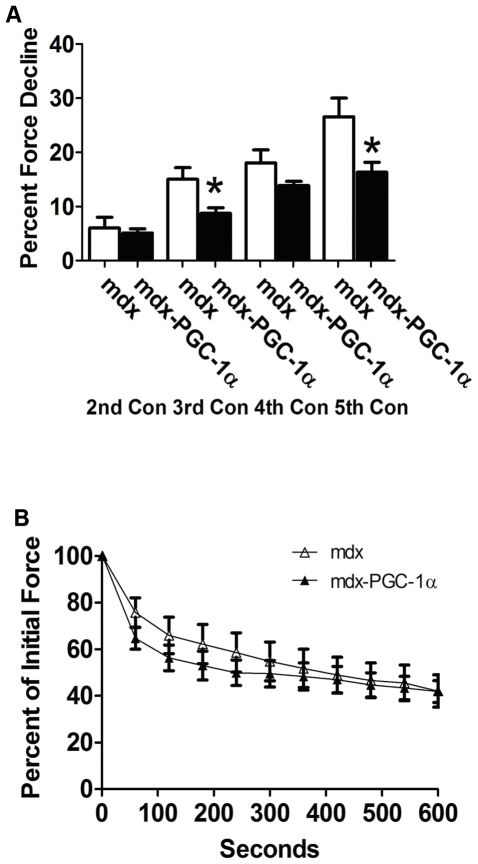
PGC-1α partially maintains diaphragmatic function six months following gene transfer. Neonatal mdx mice were injected in the sub-xyphiod region in order to cause diaphragmatic infection and sacrificed 6 mo later. Diaphragms over-expressing PGC-1α were more resistant to contraction induced injury (n = 4 Con; n = 7 PGC-1α) (A; Contraction 4 – p = 0.08), however, fatigue resistance was similar between groups (n = 7/group) (B). * indicates p<0.05.

Six months following injection of a virus causing expression of PGC-1α into the subxyphoid region, diaphragm function was assessed. As diaphragm strips may vary by size, we report only specific tension. Specific tension was similar between treated and untreated animals (Figure S6). PGC-1α over-expression improved resistance to contraction induced injury by 20–40% in contractions 3–5 (p<0.05; con 4 – p = 0.08; Figure 7), however fatigue resistance was similar between groups (Figure 7). Fibrosis was also similar between groups (Figure S7), though, there appeared to be a non-significant trend toward type I fiber expression in muscle over-expressing PGC-1α compared to control (data not shown).

### Resveratrol

**Figure 8:**
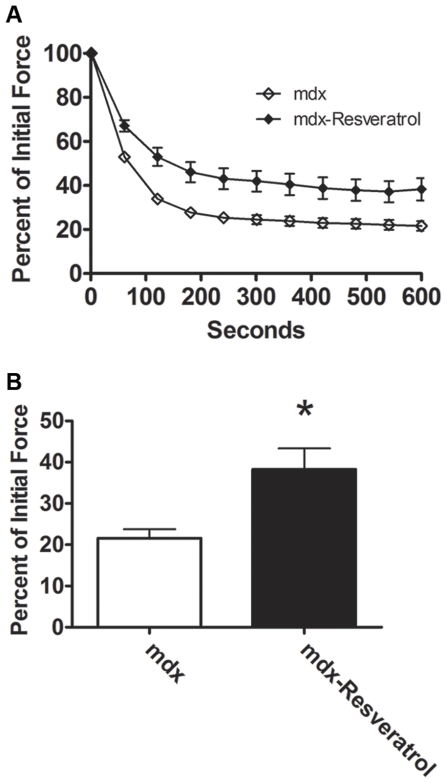
Resveratrol supplementation increased fatigue resistance in dystrophic skeletal muscle. One month old mdx mice were fed a diet containing 100 mg/kg/day resveratrol or control diet for eight weeks. Resveratrol feeding increased fatigue resistance in the soleus (A) and force generated during the final contraction (B ) was higher in treated animals when compared to control. n = 8 Con; n = 6 Res; * indicates p<0.05.

**Figure 9:**
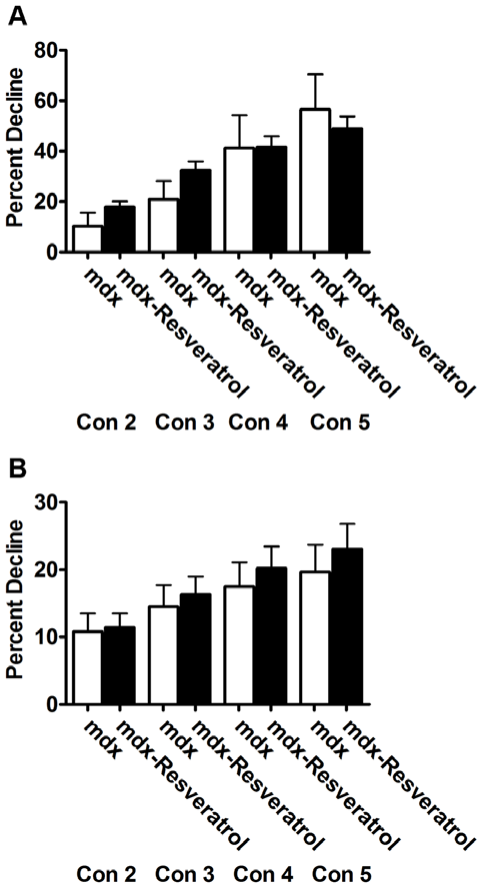
Resveratrol supplementation did not improve resistance to contraction induced injury. Feeding a diet containing 100 mg/kg/day resveratrol for eight weeks did not improve resistance to contraction induced injury in (A) the EDL (n = 8 Con; n = 6 Res) or (B) the soleus (n = 8/group) when compared to control.

To determine if an orally available Sirt-1 activator could cause similar benefits to dystrophic muscle, one month old mdx mice were fed diets containing 0 mg/kg/day, 100 mg/kg/day or 400 mg/kg/day resveratrol. Eight weeks of 100 mg/kg/day resveratrol feeding reduced body weights with a corresponding reduction in muscle mass, in similar fashion to virally induced PGC-1α (Table 3). Animals fed a diet containing 100 mg/kg/day resveratrol demonstrated increased fatigue resistance in the soleus (Figure 8), however failed to increase resistance to injury in the soleus or EDL (Figure 9). Further, elevated oxidative or slow proteins could not be detected, though utrophin expression tended to be increased in resveratrol treated animals (p = 0.13; data not shown).

**Table 3:** Muscle and body mass following eight weeks of 100 mg/kg resveratrol feeding.

	*mdx*	*mdx-Resveratrol*
Body mass (g)	33±1.3	27±1*
EDL (mg)	16.1±0.8	11.5±1.2*
Rel EDL (mg/g)	0.49±0.02	0.42±0.01*
Soleus (mg)	11.5±0.7	9.5±0.3*
Rel Soleus (mg/g)	0.25±0.01	0.35±0.01
TA (mg)	79±5	65±2*
Rel TA (mg/g)	2.4±0.1	2.4±0.05
Gastroc (mg)	191±11	149±4*
Rel Gastroc (mg/g)	5.8±0.15	5.5±0.11

*indicates significantly different from control. N = 8/group. EDL - extensor digitorum longus, Rel – relative, TA – tibialis anterior, Gastroc – gastrocnemius.

Due to the high mortality rate of the 400 mg/kg/day resveratrol group limited data was collected. Body weight and muscle mass of the soleus, gastrocnemius, TA, and EDL were similar between groups. Of potential concern was that relative heart weight was increased slightly in treated animals compared to control (4.4±0.17 mg/g Con; 5.0±0.07 mg/g; p<0.05). Muscle function in the EDL was similar between treated and control animals. In the soleus tetanic force was 25% greater (p<0.05) in treated animals compared to control (164±40 mN Control; 216±1.2 mN Resveratrol) and specific tension was increased 33% (p<0.05) in treated animals compared to control (10.3±1.2 N/cm2 Control; 15.4±1.52 N/cm2).

## Discussion

Pharmacological agents to treat Duchenne muscular dystrophy have advanced little since the late 1970s when steroid use appeared [40]. More recently an array of strategies has been proposed targeting various components of the disease [6], [7], [18], [41], [42], [43], [44]. Utrophin over-expression has repeatedly been used in animal models as an effective means of rescuing dystrophic skeletal muscle, though a practical method of human implementation has not yet been developed [18]. While it has been shown that transgenic PGC-1α over-expression would increase utrophin expression in dystrophic skeletal muscles of the mdx mouse [27], this has not been shown in the case of postnatal upregulation, which is of potential therapeutic significance. In addition, developmental abnormalities have been reported in dystrophin deficient muscle [34], [35] that could be potentially aided by transgenic PGC-1α over-expression. Hence, it is critical to establish that PGC-1α over-expression following uterine development will reduce disease severity.

These data represent the first evidence that activation of the PGC-1α pathway in post-natal dystrophic (mdx) mice will induce expression of proteins associated with the slow gene program. Consistent with what had been shown in dystrophic skeletal muscle taken from transgenic animals over-expressing PGC-1α [27], utrophin expression was elevated in treated limb muscles. Further, the increase in utrophin expression occurred in muscle with fewer centralized nuclei meaning increased utrophin expression in treated limbs was over and above that seen resulting from typical regeneration-mediated utrophin expression [23]. We also established that type I MHC is over-expressed, indicating a shift from fast-twitch (type II) to slow-twitch (type I) fibers. In healthy and dystrophic muscle Type I fibers have greater utrophin expression than type II fibers [26], [45]. Further supporting a type I shift was a reduction in muscle mass, though corresponding reductions in muscle fiber cross sectional area were not detected likely due to the high variability of fiber size in this study.

Muscle from limbs over-expressing PGC-1α was better able to maintain force production during a fatigue protocol when compared to control muscle reflecting an improved endurance capacity. Accordingly, expression of the mitochondrial proteins cytochrome C, UCP-1, and complex IV (subunit IV) as well as myoglobin were increased in PGC-1α over-expressing limbs compared to control limbs. Given the improved endurance capacity and increased expression of oxidative proteins, it is likely that the cells are more capable of ATP production and may also indicate an increased mitochondrial number or volume. Speculatively, aside from an increased platform to produce ATP, increased mitochondrial volume is likely beneficial to dystrophic muscle as a secondary well for Ca^2+^ sequestering [12], [14] potentially limiting the contributions of calpains [5], [6], [46] and free radicals [8], [9], [10] to disease-related muscle injury. Further, as a consequence of increased mitochondrial volume we would hypothesize that there is less Ca^2+^/mitochondrion leading to less mitochondrial dysfunction throughout the cell providing an additional means for increased ATP production. The metabolic aspect of dystrophin-deficiency is an important consideration as it appears very early in disease progression [47], [48] and may represent a primary mechanism of disease pathology.

In addition to measuring slow and oxidative protein expression we also assessed PGC-1α pathway changes as a result of PGC-1α over-expression. Interestingly, Sirt-1 expression was increased in treated muscle when compared to control despite being upstream of PGC-1α. Sirt-1 is a deacetylase that acts on PGC-1α to increase its activity [36], [37], [38]. It seems likely that Sirt-1 activity could lead to either oxidative or slow gene activation. In contrast, phosphorylated p38 and NRF-1 content were reduced in PGC-1α over-expressing muscle. Long-term reduction in NRF-1 expression may help to explain the observation that resistance to fatigue is similar in treated and control 6 mo old diaphragms. It is possible that this observation is an attempt to decrease signaling through the PGC-1α pathway in the face of massive PGC-1α over-expression and that NRF-1 and phosphorylation of PGC-1α by p38 represent points of regulation. Alternatively, reduced phospho-p38 expression may also indicate that cells from treated limbs are under less stress than cells from control limbs [49], [50].

For the potential benefits of PGC-1α to be evaluated in human DMD patients, a pharmacological approach must be identified. Previous studies using resveratrol to activate Sirt-1 (a PGC-1α activator) demonstrated increased oxidative gene expression [36], [51]. Activation of this pathway has protected mice against diabetes related pathologies as well as helped to prevent obesity [36], [51]. Thus, in an independent experiment, mdx mice were fed 100 mg/kg resveratrol or control diet for two months. Supplementation of the diet with resveratrol resulted in improved fatigue resistance and reduced muscle weights, which is consistent with observations made in comparing PGC-1α over-expressing limbs to control limbs. It did not, however, improve resistance to contraction-induced injury though tended to increase utrophin content. Thus, resveratrol mimicked some, but not all, changes associated PGC-1α pathway activation suggesting that either an alternative dose of resveratrol or more potent Sirt-1 activator [39] should be evaluated.

An alternative approach is to provide orally available PPAR agonists. Indeed, a PPAR agonist was recently used to increase utrophin expression and reduce eccentric muscle injury in mdx mice [52]. Caution should be taken with use of these drugs, however, as PPAR agonists known as thiazolidinediones have recently been linked to heart disease [53], [54], [55] and cancer [56]. Related, our initial study design called for an mdx group to be supplemented with 400 mg/kg/day, however, despite early replacement of lost animals, only three animals reached the end of the eight week dosing regimen indicating potential toxicity with this drug. Given though the success of other investigations at this dose it may point to a source dependent effect rather than an effect of the resveratrol dose, per se.

In summary, activation of the PGC-1α pathway could lead to dystrophic muscles that are more resistant to contraction induced damage and fatigue. Further, we demonstrated that the basis of improved muscle function likely involves a shift to slower fiber types with concomitant increases in utrophin expression. In the dystrophic skeletal muscles of humans with DMD, type II fibers have been shown to undergo degeneration before slow fiber types, providing evidence in humans of the protective effects of the slow gene program, and likely increased utrophin levels [57]. Moreover, it has been shown that variability in disease severity in DMD boys is linked to differences in utrophin expression levels [58]. It is reasonable to suggest that this may reflect differences in the percentage of slow vs. fast fibers among individual DMD patients. If so, then the approach of shifting human DMD muscles toward an increased slow-twitch fiber profile may indeed provide therapeutic benefit. However, the degree of benefit seen in the mdx mouse is likely to be much greater since it begins with higher fast fiber type content than humans. Future studies will attempt to ascertain how much benefit is tied to the possible fiber type shift and the impact on both slow and fast-twitch muscles needs to be differentially evaluated.

## Methods

### Animal treatments

All animals procedures were done in accordance with the guiding principles of animal use established by the American Physiological Society and were approved the IACUC at the University of Pennsylvania (protocol number 801770). In order to test our hypotheses, neonatal mdx mice from our colony were injected with adeno-associated virus (AAV) pseudotype 2/6 expressing mouse PGC-1α. To inject pups, a technique called cryosedation was used. Briefly, pups were placed on ice with a thin plastic barrier until they could be handled without excessive movement, allowing accurate and precise injection [59]. In one investigation, mice were injected with 1×10^11^ gc delivered in 50 ul to the lower right hind limb with a depth of approximately 1 mm while contralateral limbs were injected with an equal volume of saline [44], [60], [61]. This technique is effective for achieving transgene expression in the soleus, gastrocnemius, tibialis anterior, and extensor digitorum longus. Mice were sacrificed at four and six weeks of age. For long-term studies, neonatal mice were injected with virus as above in the subxyphoid region in order to infect the diaphragm or given a sham injection and sacrificed at six months of age [44], [62].

In an independent study designed to determine the extent to which an orally available Sirt-1 activator could mimic the effects of virally mediated PGC-1α over-expression, one month old male mdx mice were fed either a control diet (AIN-93; Bioserv) or a diet containing 100 mg/kg/day of resveratrol (Sigma) for eight weeks. We also included a group of animals that was given 400 mg/kg/day resveratrol. Only 3 treated animals in this group survived the eight week study period, despite early efforts to replace several lost animals, hence only limited data was collected.

To prepare virus, PGC-1α cDNA was amplified from mouse skeletal muscle mRNA using primers that added restriction sites at the 5′ and 3′ ends. The restriction sites allowed the PGC-1α transgene to be inserted into an expression vector developed at the Vector Core at the University of Pennsylvania designed to express PGC-1α under control of the constitutive chicken beta actin promoter with CMV enhancer. After confirming the sequence of the insert and inverted terminal repeats (ITR) in the plasmid it was amplified for viral preparation. Vectors were produced according to the previously described pseudotyping protocol by the Vector Core at the Children's Hospital of Philadelphia [63]. Briefly, recombinant AAV genomes containing AAV2 ITR's were packaged by triple transfection of 293 cells with a cis-plasmid containing the PGC-1α transgene, an adenovirus helper plasmid, and a chimeric trans-plasmid containing the AAV2 rep gene fused to the capsid gene of the AAV6 serotype. AAV6 has been previously shown to successfully deliver transgenes into skeletal muscle [64].

### Muscle function

Upon animal sacrifice, muscle mechanics testing was performed on muscles removed from the mice at the Physiological Assessment Core of the Wellstone Muscular Dystrophy Cooperative Center at the University of Pennsylvania. All animals were given an injection of a ketamine/xylazine cocktail to induce a surgical level of anesthesia. Limb muscles or diaphragms were removed as appropriate, weighed, and contractile function was performed according to standard techniques [6], [44], [62], [65], [66]. Briefly, function was determined with an Aurora dual mode lever system (Ontario, Canada) interfaced with a Dell Dimension 2400 desk top computer and controlled with DMC software (version 3.2). Muscles were placed in oxygenated Ringers solution such that the proximal and distal tendons were attached to clamps. One end was connected to a force transducer and the other to an anchor. Bilateral electrodes were placed longitudinally adjacent to the muscle to create a field upon stimulation. Optimum length (Lo) was determined using standard techniques followed by supramaximal stimulation (EDL – 120 Hz, Sol – 100 Hz, diaphragm – 100 Hz) in order to achieve tetanic contractions. Each muscle performed three 500 msec tetanic contractions at Lo with five minutes between each trial. Cross sectional area (CSA) and specific tension were estimated using standard equations and constants [67]. Some EDL muscles, solei, and one diaphragm strip were given a series of five lengthening contractions in order to determine resistance to damage (80 Hz for 500 msec followed by 200 msec at 110% Lo). Other EDL muscles, solei and one diaphragm strip were stimulated for once per second for 10 minutes (200 µsec pulse, 100 Hz, 330 msec duration) in order to determine resistance to fatigue.

### Histology

Muscles were removed, weighed, and frozen in melting isopentane. Ten micrometer histological sections were cut at −30°C with a Leica CM3000 cryostat (Bannockburn, IL). Hematoxylin and eosin staining were accomplished according to standard techniques. Immunohistochemistry was performed as follows: tissues were washed in PBS for 10 minutes and blocked by covering each section with 5% BSA for 15 minutes. Sections were then incubated overnight at 4°C in primary antibody (MHCs, Nova Castra, New Castle, UK; Laminin, Neomarkers, Fremont, CA) at a dilution of 1∶100 in 5% BSA. They were then washed three times in PBS and incubated with secondary antibody at a dilution of 1∶200 in 5% BSA for one hour in the dark. Sections were washed three times for ten minutes in PBS and the coverslip mounted with Vectashield with DAPI. In order to determine the extent to which central nucleation was altered by our intervention the total number of cells in a cross section was determined (approximately 1,000 cells). Next, muscle cells containing a centralized nucleus were counted. Data is expressed as the percent of total muscle cells counted with a centralized nucleus. To measure the cross sectional area of muscle fibers the cross sectional area of each muscle fiber in a section was measured using calibrated Openlab software. The cross sectional area of each fiber was recorded then assigned to a size range (binning). The mean percentage of fibers within a bin was determined for each size range for both untreated and treated limbs.

### Biochemistry

Selected muscles were also removed, weighed and frozen in liquid nitrogen for biochemical analysis. Western blotting was done as previously described with minor modifications [68]. Muscle was powdered on dry ice and lysed using lysis buffer at a 1∶10 dilution [6]. Protein concentration was determined by the method of Biuret. Following dilution of whole homogenate to 2.5 mg/ml in reducing buffer, 25 µg protein was loaded into 4–20% gradient gels and separated vertically by molecular weight. Transfer was accomplished by using the I-blot system from Invitrogen (Carlsbad, CA). Primary antibodies were used as described below in 1% milk dissolved in 0.1% Tween-20 buffered TBS overnight at 4°C. Primary antibodies were used as follows: utrophin 1∶100 (Vector – Burlingame, CA), cytochrome C 1∶1000 (Santa Cruz, Santa Cruz, CA), TnI 1∶1000 (Santa Cruz), Hsp 60 1∶1000 (Abcam – Cambridge, MA), MHCs 1∶1000 (Nova Castra), Sirt-1 1∶1000 (Millipore – Bedford, MA), phospho-p38 1∶1000 (Cell Signaling Technology – Boston, MA), p38 1∶1000 (Cell Signaling Technology), UCP-1 1∶100 (Abcam), Complex IV subunit IV 1∶2500 (Mitosciences – Eugene, OR), NRF-1 1∶000 (Abcam), myoglobin 1∶500 (Santa Cruz), actin 1∶2000 (Neomarkers), and spectrin 1∶1000 (Novacastra). Secondary antibodies were incubated for one hour at room temperature at 2× primary dilution such that an antibody used at 1∶1000 for primary antibody would receive a 1∶2000 dilution of secondary in 1% milk dissolved in TTBS. Blots were exposed to SuperSignal (Thermo Scientific, Rockford, IL) for 3–4 minutes and emitted light captured with film. Following development, blots were quantified using Kodak software (Rochester, NY) with the automated band find function, where possible, in order to remove researcher bias. Further, beta-actin was quantified and used as a loading control to assure equal amounts of protein were loaded for each sample. Actin did not differ between groups for any gel run indicating consistent loading.

RNA was isolated according to standard techniques. Briefly, muscle was powdered over dry ice and homogenized with Trizol. Following centrifugation and extraction with chloroform, RNA was precipitated with isopropanol. The RNA pellet was resuspended in nuclease free water. rtPCR was performed to convert RNA to cDNA, and then qPCR was used to assess gene expression. Two sets of primers were designed to distinguish between total and exogenous PGC-1α. The first, which measured total PGC-1α, was designed internally within the gene. The second set, which was used to determine viral expression of PGC-1α, was directed against the viral 3′ UTR.

### Statistics

Data collected as part of PGC-1α gene transfer studies, where control and treated limbs are found within the same animal, were compared using a paired T-test. Data collected as part of resveratrol supplementation studies were compared using a T-test. Resistance to fatigue was determined by comparing force generated during the final contraction. Significance was determined a priori at p<0.05. Data are presented as means ± SEM unless otherwise noted.

## Supporting Information

Figure S1 **PGC-1α over-expression fails to improve resistance to contraction induced injury in the soleus.** Six weeks following viral injection of a virus driving PGC-1α, treated solei had a similar resistance to contraction induced injury as control muscle. n = 7/group.
(PDF)

Figure S2 **Resistance to fatigue is improved with PGC-1α gene transfer.** Four weeks following injection of virus driving PGC-1α, soleus muscles were more resistant to fatigue than control. The fatigue curve (A) was significantly different from control and the force generated during the final contraction (B) was higher in PGC-1α over-expressing muscle when compared to control. N = 7/group; * indicates p<0.05.
(PDF)

Figure S3 **Muscle mass and fatigue resistance were poorly correlated.** The percent of initial force from fatigue curves was plotted against muscle mass in order to determine the extent to which muscle size may impact fatigue data. As four and six week old mdx hind limb muscle are physiologically distinct and treated and control limbs are physiologically distinct they were fitted independently. R^2^ and corresponding p-values were as follows: 4 wk mdx – 0.006, p<0.86; 4 wk mdx-PGC-1α – 0.055, p<0.61; 6 wk mdx – 0.07, p<0.61; 6 wk mdx-PGC-1α – 0.42, p<0.15.
(PDF)

Figure S4 **Fiber area distribution in the soleus 6 wks following gene transfer.** The percent of fibers within a given cross sectional area range was determined for treated and untreated soleus muscles (approximately 1,000 fibers/muscle). Fiber area distribution was similar between groups. n = 5/group.
(PDF)

Figure S5 **Protein expression in the soleus 6 wks following gene transfer.** Representative Western blots from the soleus (A) were quantified and generally support the notion of increased utrophin (B) and expression of oxidative proteins (C and D). n = 9/group; * indicates P<0.05.
(PDF)

Figure S6 **Specific tension in the diaphragm six months following gene transfer.** Six months of PGC-1α over-expression (n = 7) did not improve the specific tension in diaphragm strips compared to control muscle (n = 8).
(PDF)

Figure S7 **Fiber and Fibrotic area in the diaphragm.** Six months following PGC-1α over-expression, fibrotic area and fiber area were similar between treated and untreated animals (n = 6/group).
(PDF)
