# Supplementary figures and images for: Rescue of Dystrophic Skeletal Muscle by PGC-1α Involves a Fast to Slow Fiber Type Shift in the mdx Mouse
Source: PLoS One. 2012 Jan 11;7(1):e30063. doi: 10.1371/journal.pone.0030063 (PMC3256197; doi:10.1371/journal.pone.0030063)

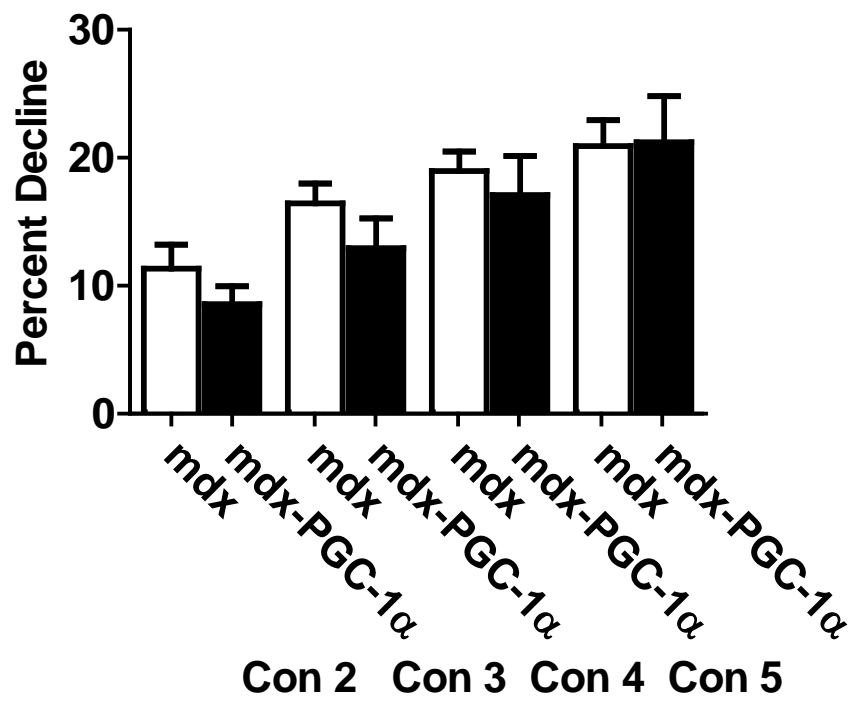

Supplement: Figure S1 — PGC-1α over-expression fails to improve resistance to contraction induced injury in the soleus. Six weeks following viral injection of a virus driving PGC-1α, treated solei had a similar resistance to contraction induced injury as control muscle. n = 7/group. (PDF) [file pone.0030063.s001.pdf]

A

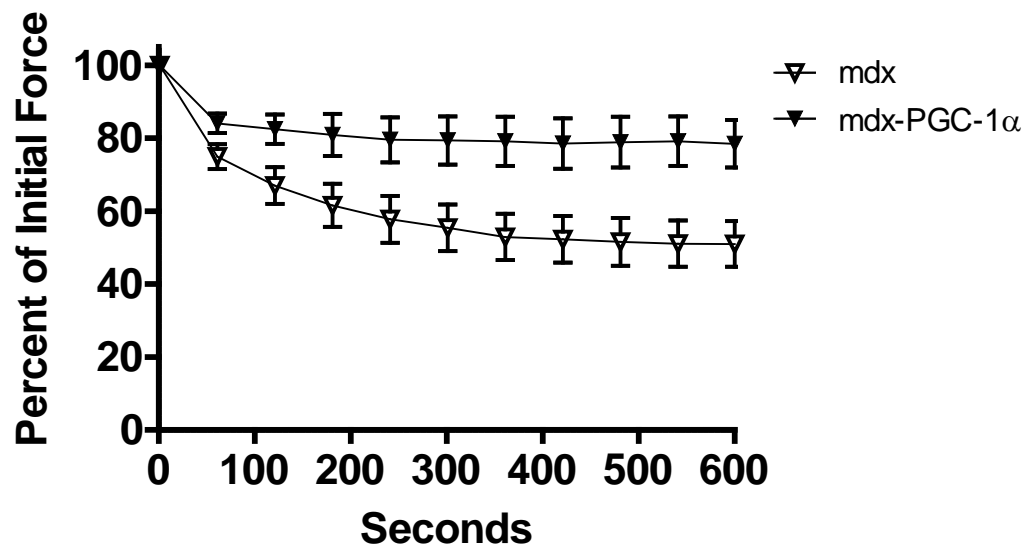

B

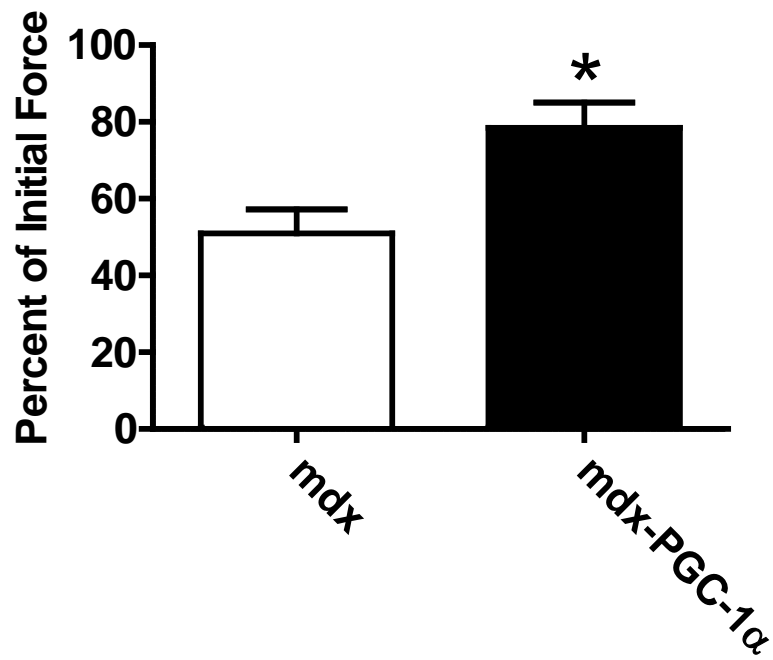

Supplement: Figure S2 — Resistance to fatigue is improved with PGC-1α gene transfer. Four weeks following injection of virus driving PGC-1α, soleus muscles were more resistant to fatigue than control. The fatigue curve (A) was significantly different from control and the force generated during the final contraction (B) was higher in PGC-1α over-expressing muscle when compared to control. N = 7/group; * indicates p<0.05. (PDF) [file pone.0030063.s002.pdf]

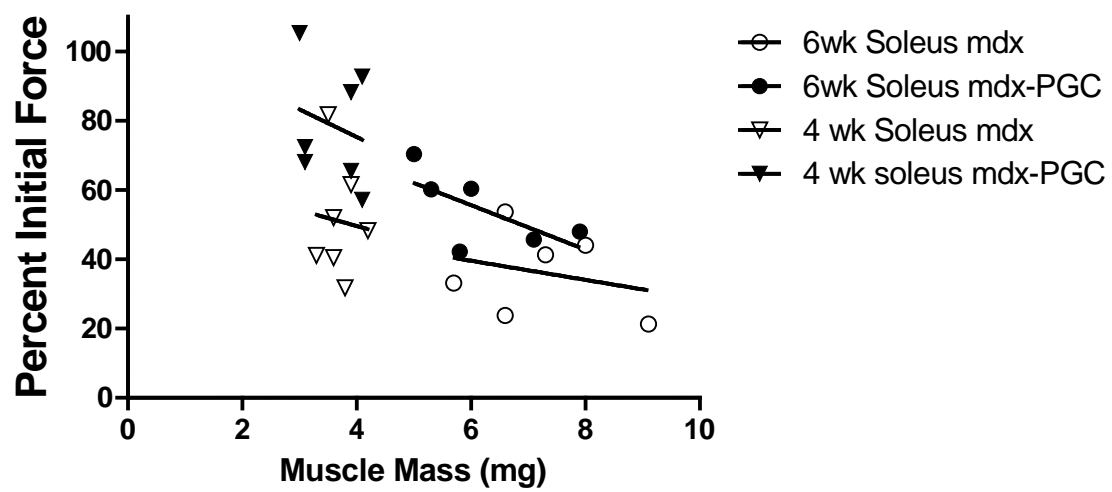

Supplement: Figure S3 — Muscle mass and fatigue resistance were poorly correlated. The percent of initial force from fatigue curves was plotted against muscle mass in order to determine the extent to which muscle size may impact fatigue data. As four and six week old mdx hind limb muscle are physiologically distinct and treated and control limbs are physiologically distinct they were fitted independently. R2 and corresponding p-values were as follows: 4 wk mdx – 0.006, p<0.86; 4 wk mdx-PGC-1α – 0.055, p<0.61; 6 wk mdx – 0.07, p<0.61; 6 wk mdx-PGC-1α – 0.42, p<0.15. (PDF) [file pone.0030063.s003.pdf]

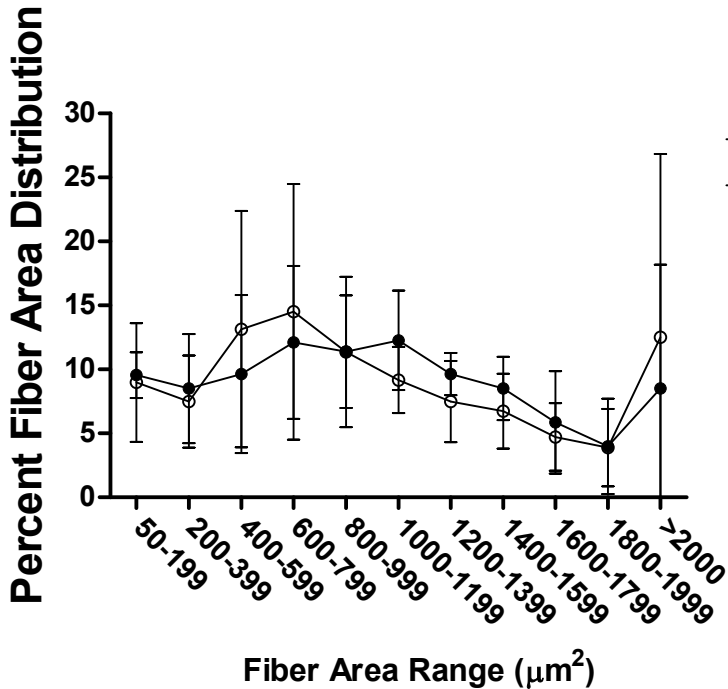

Supplement: Figure S4 — Fiber area distribution in the soleus 6 wks following gene transfer. The percent of fibers within a given cross sectional area range was determined for treated and untreated soleus muscles (approximately 1,000 fibers/muscle). Fiber area distribution was similar between groups. n = 5/group. (PDF) [file pone.0030063.s004.pdf]

A

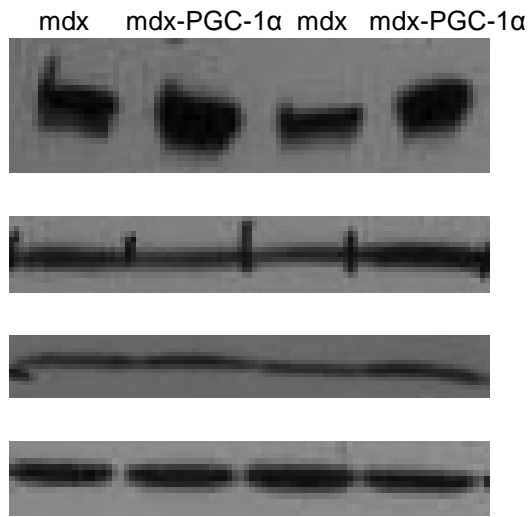

Utrophin

Cytochrome C

Myoglobin

Actin

B

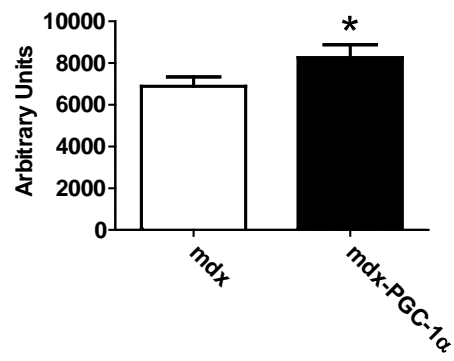

C

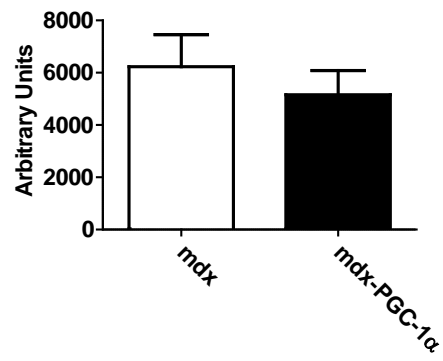

D

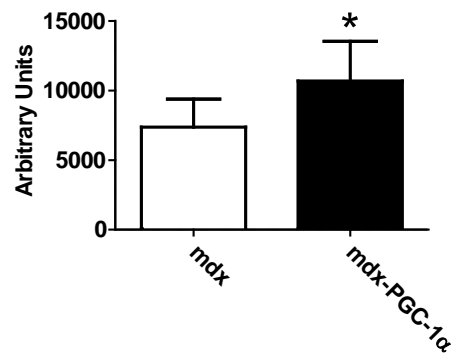

Supplement: Figure S5 — Protein expression in the soleus 6 wks following gene transfer. Representative Western blots from the soleus (A) were quantified and generally support the notion of increased utrophin (B) and expression of oxidative proteins (C and D). n = 9/group; * indicates P<0.05. (PDF) [file pone.0030063.s005.pdf]

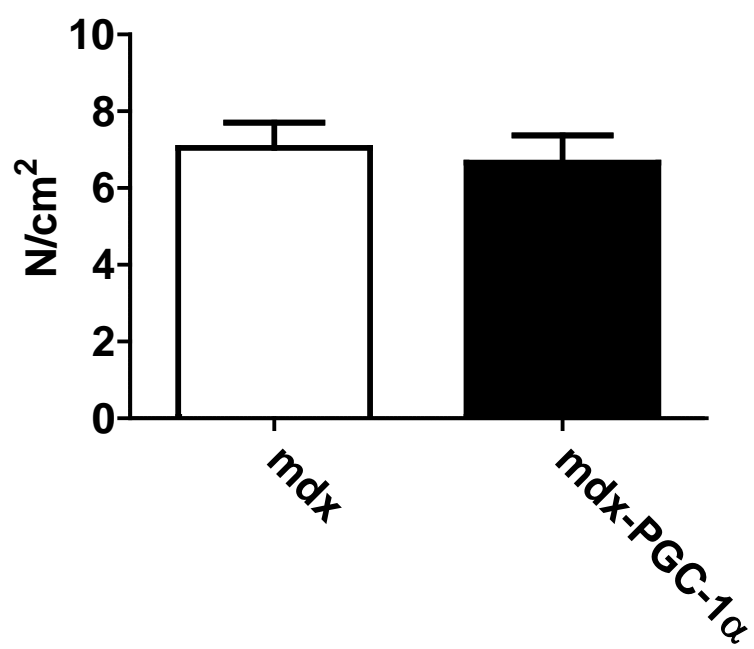

Supplement: Figure S6 — Specific tension in the diaphragm six months following gene transfer. Six months of PGC-1α over-expression (n = 7) did not improve the specific tension in diaphragm strips compared to control muscle (n = 8). (PDF) [file pone.0030063.s006.pdf]

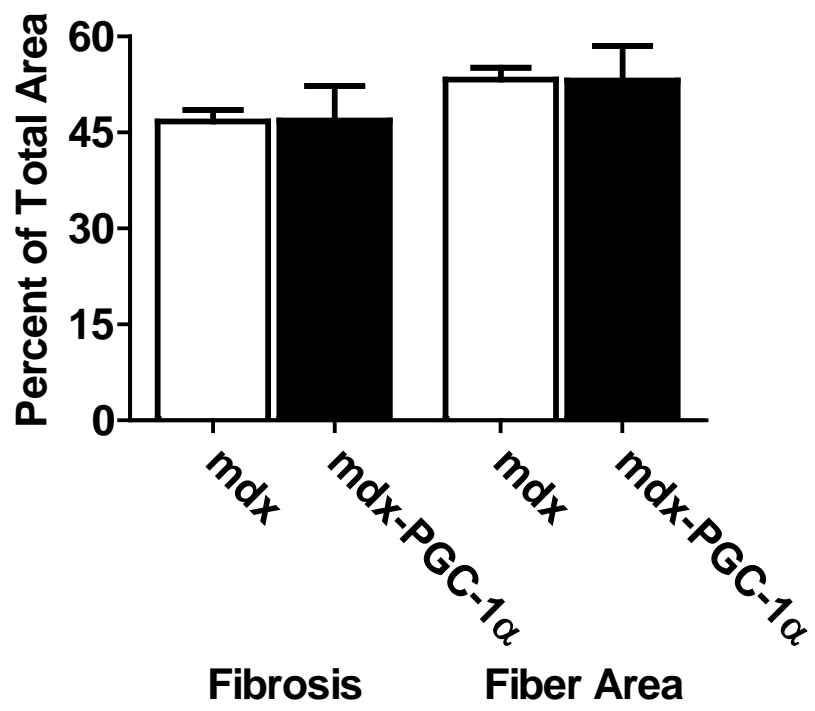

Supplement: Figure S7 — Fiber and Fibrotic area in the diaphragm. Six months following PGC-1α over-expression, fibrotic area and fiber area were similar between treated and untreated animals (n = 6/group). (PDF) [file pone.0030063.s007.pdf]
